# Genome Sequence of a Heterocystous Diazotroph Isolated from a *Trichodesmium* Consortium from the North Pacific Subtropical Gyre

**DOI:** 10.1128/mra.00453-22

**Published:** 2022-12-12

**Authors:** Samantha Lei, Elaina D. Graham, John F. Heidelberg, Eric A. Webb

**Affiliations:** a Department of Biological Sciences, University of Southern California, Los Angeles, California, USA; b Department of Biological Sciences, Marine Environmental Biology Section, University of Southern California, Los Angeles, California, USA; Montana State University

## Abstract

Diazotrophic cyanobacteria play a vital role in the nitrogen influx of the global marine ecosystem. In July 2010, colonies of *Trichodesmium* spp. were picked near Station ALOHA in the oligotrophic North Pacific Subtropical Gyre, and a novel heterocystous diazotroph (strain HetDA_MAG_MS3) belonging to the genus *Rivularia* was found living in close association; it was cultured and sequenced.

## ANNOUNCEMENT

Here, we describe a genome for a heterocystous diazotrophic cyanobacterium, strain HetDA_MAG_MS3. Initial samples were collected as detailed by Momper et al. (1), by using microscopy to pick *Trichodesmium* colonies and incubating them in sterile YBC-II medium at 24°C with a 12-h light (100 μmol photons m^−2^ s^−1^)/12-h dark cycle. The enrichment was grown under the aforementioned conditions for 5 years prior to sequencing and was transferred to fresh medium every month. A 50-mL subsample of the enrichment was gravity filtered onto a 5.0-μm polycarbonate filter, and DNA was extracted using the Qiagen DNeasy PowerSoil kit. Extracted DNA was sent to the University of Southern California Epigenome Center and sequenced on an Illumina MiSeq sequencer (paired-end 250-bp reads, with a total of 4,725,335 raw reads) using the MiSeq reagent kit v2 with 300 cycles. Reads were trimmed using Trimmomatic v0.38 (parameters: –phred33, ILLUMINACLIP:TruSeq3-PE.fa:2:30:10 SLIDINGWINDOW:10:28 MINLEN:50) (2), assembled using MetaSPAdes v3.14.0 (3), and mapped to the assembly using Bowtie2 v2.3.5 (4), and the mapping results were filtered using CoverM v0.4.0 (parameters: –min-read-percent-identity 0.95 –min-read-aligned-percent 0.75) (5). The assembly was binned using MetaBat2 v2.12.1 (6), BinSanity-wf v0.3.8 (7), and Concoct v1.1.0 (8) with default parameters for each, and DASTool v1.1.2 (9) was used to determine a final set of metagenome-assembled genomes (MAGs). Following this, all bins were run through MetaSanity (10), PhyloSanity, and FuncSanity for annotation. One of these draft genomes, which we refer to as HetDA_MAG_MS3, was classified by GTDB-Tk (11) as an unnamed cyanobacterium belonging to the *Nostocaceae* family and the *Rivularia* genus, with an average nucleotide identity (ANI) value of 89.58% with respect to GenBank assembly accession number GCA_002471275.1. The *nifH* gene found in this *Rivularia* MAG matches that of the HetDA genus found in previous work by Momper et al. (1). The genome was determined to be 99.33% complete, with a GC content of 37.3%, as assessed using CheckM (12). The genome has 9,193,454 bp and 174 contigs, with an *N*_50_ value of 93,019 bp. The genome has 7,301 predicted genes and a coding density of 78.63%.

Based on the presence of the active form 1 ribulose-1,5-bisphosphate carboxylase-oxygenase (RubisCO) (KEGG Orthology code K01601) and the genes required for the Calvin cycle, HetDA_MAG_MS3 is an aerobic oxygenic photoautotroph (13). The genome also contains the genes for photosystems II and I, ATPase, and many of the other proteins required for the electron transport chain (14), further supporting its autotrophic lifestyle. Like other marine organisms living near the surface, this genome also contains one-half of the retinal biosynthesis genes (KEGG Orthology codes K02291 and K13789), which are important in protecting the cells from wavelengths of light the organism cannot absorb, but does not contain rhodopsin.

This MAG contains *nifHDK* (KEGG Orthology codes K02586, K02588, and K02591), indicating nitrogen-fixing capabilities. Its closest relative is *Rivularia* strain PCC 7116 (GenBank assembly accession number GCF_000316665.1), as determined by GTDB-Tk. Fitting with the morphology of HetDA, the *Nostocales* family is known for including genera that form heterocysts (15). HetDA_MAG_MS3 has the potential for sulfur assimilation via sulfite reductase (ferredoxin) (KEGG Orthology code K00392) and sulfide oxidation to polysulfide via sulfide-quinone oxidoreductase (KEGG Orthology code K17218). These intermediates could act as the substrates for other microbial processes (16).

In summary, HetDA_MAG_MS3 is an aerobic oxygenic photoautotroph with the potential for nitrogen fixation and sulfur oxidation.

### Data availability.

Raw sequences and genomes are available under BioProject accession number PRJNA719568. Raw reads were deposited in the SRA under accession number SRR14140256. The genome is available under BioSample accession number SAMN18613313.
